# Scar quality in children with burns 5–7 years after injury: A cross‐sectional multicentre study

**DOI:** 10.1111/wrr.12953

**Published:** 2021-06-16

**Authors:** Inge Spronk, Anniek Stortelers, Cornelis H. van der Vlies, Paul P. M. van Zuijlen, Anouk Pijpe, Burden of Burn Injuries Study Group

**Affiliations:** ^1^ Association of Dutch Burn Centres Maasstad Hospital Rotterdam Netherlands; ^2^ Department of Public Health Erasmus MC, University Medical Center Rotterdam Rotterdam Netherlands; ^3^ Burn Centre Red Cross Hospital Beverwijk Netherlands; ^4^ Burn Centre Maasstad Hospital Rotterdam Netherlands; ^5^ Trauma Research Unit Department of Surgery Erasmus MC, University Medical Center Rotterdam Rotterdam Netherlands; ^6^ Dept. of Plastic, Reconstructive & Hand Surgery, Amsterdam Movement Sciences Amsterdam UMC, Vrije Universiteit Amsterdam Amsterdam Netherlands; ^7^ Department of Plastic Reconstructive and Hand Surgery, Red Cross Hospital Beverwijk Netherlands; ^8^ Paediatric Surgical Centre Emma Children's Hospital, Amsterdam UMC Amsterdam Netherlands

**Keywords:** burn injuries, children, long‐term outcomes, scar quality

## Abstract

Long‐term scar formation is an important adverse consequence in children with burns, however, information regarding scar quality in the long‐term is lacking. Therefore, we evaluated scar quality and its predictors in children with burns 5–7 years after injury. Parents of children with mild/intermediate burns (≤10% total body surface area burned), and of children with severe burns (>10% burned) completed the patient scale of the Patient and Observer Scar Assessment Scale (POSAS 2.0) for their children's—in their opinion—worst scar 5–7 years post‐burn. Outcomes and predictive factors of scar quality were studied, and, for children with severe burns, POSAS parent scores were compared with observer scores. We included 103 children with mild/intermediate burns and 28 with severe burns (response rate: 51%). Most children (87%) had scars that differed from normal skin, with most differences reported for colour, and least for pain. Except for colour, children with severe burns had significantly higher scores (difference 0–2 points) on all scar characteristics (representing poorer scar quality) compared with children with mild/intermediate burns. Parent POSAS scores were on average 2.0–2.6 points higher compared to observer scores. Number of surgeries predicted both the mean POSAS and the mean overall opinion of a scar. In conclusion, 5–7 years post‐burn, the scar of the majority of children differed from normal skin, especially on the characteristic colour. The uncovered insights are useful in counselling of children and their parents on expectations of the final outcome of their (children's) scar(s), and help in further targeting scar prevention strategies for the individual child.

**Abbreviations**
IQR: interquartile range
POSAS: Patient and Observer Scar Assessment Scale
TBSA: total body surface area

## 1 INTRODUCTION

A burn injury suddenly disrupts a child's life. Burns may hamper children's physical, psychological and social wellbeing, as well as their families' wellbeing.^1^, ^2^, ^3^ To understand and quantify the consequences of burn, investigating outcomes of treatment and rehabilitation is gaining interest.^3^ Scar formation is an important adverse consequence of burns. Paediatric scars can cause long‐term disfigurement, as well as physical and psychological problems, and may result in a diminished health‐related quality of life.^2^ Assessment of patient perspectives is important as they may differ from clinician perspectives.^4^, ^5^ Patients have to live with their scars and by assessing the severity of their own scar(s), therapy is more likely to fit to their needs.^6^

Studies on scar quality in children with burns have been performed; however, scar quality was only assessed up‐to 28 months after injury.^4^, ^7^, ^8^, ^9^, ^10^ Research on scar quality in the longer term is lacking. Studies showed that most change in scar quality is seen in the first months after burn injury.^7^, ^8^ Several factors have been reported to predict paediatric scar quality, including time to wound healing, a greater percentage total body surface area (%TBSA) burned, full thickness wounds, and multiple surgeries.^4^, ^8^, ^9^, ^10^, ^11^, ^12^, ^13^, ^14^ However, it is also known that scar maturation can take several years,^15^ and therefore, it is important to examine scar quality and associated risk factors in the longer term. Therefore, the aim of this study was to evaluate scar quality and its predictors in children with burns 5–7 years after injury, separately for children with mild/intermediate burns and severe burns.

## 2 METHODS

This study is part of the Burden of Burn Injuries study; a cross‐sectional study on long‐term consequences of burns.^16^ This study was conducted according to the principles of the Declaration of Helsinki, and approved by the Ethics Committee (NL59981) and the institutional review boards of the three participating hospitals. The study was registered at the Netherlands Trial Register (NTR6407). A written informed consent form was signed by both parents, and by the child if ≥12 years old.

### 2.1 Study population

Children (<18 years old at study) admitted in a Dutch burn centre between 08/2011 and 09/2012 were selected from the Dutch Burn Repository R3.^17^ As this is a 5–7 year follow‐up study, we included children <13 years old at burn injury. The rationale for this selection is that children up to age 18 are treated as children in Dutch health care.

As only a limited number of Dutch paediatric burn patients have severe burn injuries (about 22 each year), we extended this sample with children with severe burns (>10% TBSA burned if aged <10 years old at burn, >20% TBSA if aged ≥10 years old, or more than 5% full thickness burns^18^) admitted between 01/2010 and 03/2013 to elucidate scar quality after severe burns. Patients were not eligible when parents were unable to answer the questionnaires, the patient was deceased, or when contact details were missing.

### 2.2 Study procedure

Parents of eligible children were invited to participate through a postal letter containing an information leaflet, an informed consent form and the first survey. A second survey was sent after receiving informed consent, and children with severe burns were invited for scar assessment and physical measures at the outpatient clinic. Surveys were filled in by a parent(s). If no response was obtained, parents were called after 3 weeks to discuss participation. A postal reminder was sent if parents were not reached by telephone or did not return the informed consent form and survey after promising they would.

### 2.3 Study parameters

The first survey included the patient scale of the Patient and Observer Scar Assessment Scale (POSAS) 2.0.^19^ This scale assesses six scar characteristics: pain, itching, colour, pliability, thickness and relief, and includes an overall opinion item. Parents completed the POSAS for their child's—in their opinion—worst scar. We defined scars located on the hands/arms/feet/legs/face/neck as functional site scars. Scars on hands/face were indicated as visible scars. The six characteristics were scored between 1 and 10, with a score of 1 representing ‘no differences with normal skin’ and a score of 10 ‘very different to normal skin’. The POSAS score was calculated by summing up the six scores and dividing this by six. In case five of the six characteristics were completed, the POSAS score was calculated based on these five scores. The overall opinion item was measured on a 10‐point scale, with 1 meaning ‘as normal skin’ and 10 ‘worst scar imaginable’.^20^

The POSAS also includes an observer scale.^20^ For children with severe burns visiting the outpatient clinic, two experienced and trained observers independently assessed the worst scar. The mean score of the observers was used as the observer score. Colour, thickness, relief and pliability scores of observers and parents were compared. A mean POSAS observer score was calculated by summing up the mean score of these four characteristic scores and dividing this by four. The same was done for the parent scores.

Other study parameters were extracted from the Dutch burn repository^17^ and included patient characteristics (age, gender); burn characteristics (%TBSA burned, % full thickness burns, aetiology); and clinical characteristics (date of injury, number of surgeries, length of hospital stay, reconstructive surgery, artificial ventilation).

### 2.4 Statistical analyses

A non‐response analysis was done to compare children of participating parents with children of non‐participating parents. All variables were checked for normality. If variables were not normally distributed, the median as well as the interquartile range (IQR) was reported. Continuous variables were compared with Mann–Whitney *U*‐tests and categorical variables with chi‐square tests, except for small numbers (*n* < 5), then the Fisher's exact test was used.

Long‐term scar quality, including the six POSAS items, the POSAS score, and the overall opinion score, was assessed using descriptive statistics. Outcomes of children with mild/intermediate burns (<10% TBSA burned) were compared with children with severe burns (≥10% TBSA burned, or > 5% full thickness burns), and, parent scores were compared against observer scores.

To identify predictive factors of scar quality, univariate linear regression was used. Burn centre dependency was tested in a mixed model analysis. None of the univariate analyses were centre dependent; therefore, we used linear regression analysis. All variables with a *p*‐value <0.10 in univariate analyses were checked for collinearity (>0.8 or <−0.8) and included in multivariate linear regression. A *p*‐value of <0.05 was considered statistically significant. Analyses were performed with IBM SPSS Statistics 25.

## 3 RESULTS

### 3.1 Participants

**FIGURE 1:**
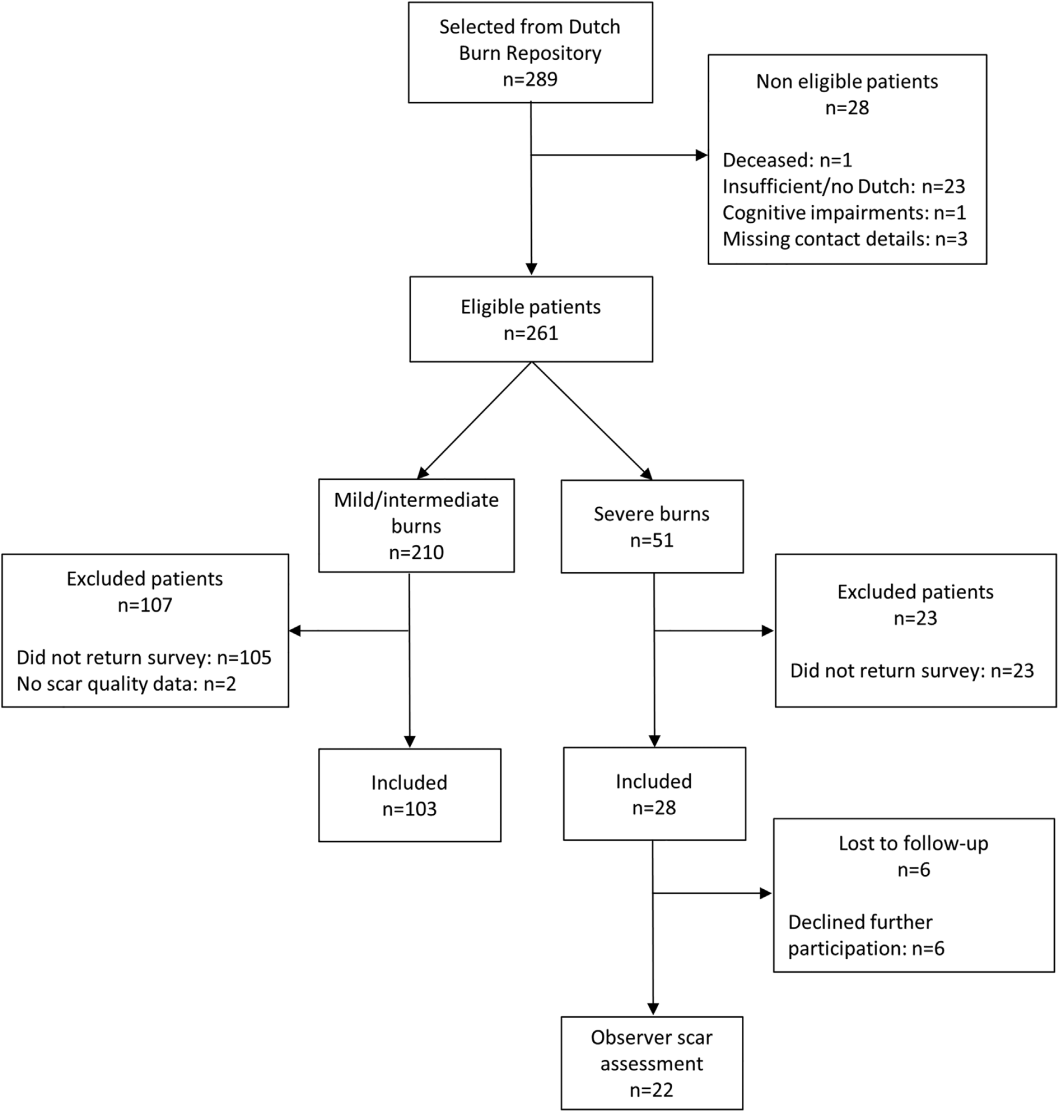
Flowchart inclusion of patients

In total, 289 children were selected, of whom 261 were eligible (Figure 1). Half of the parents (*n* = 131) participated; including parents of 103 children with mild/severe burns (response rate: 49%) and 28 children with severe burns (response rate: 55%). Children of non‐responders did not differ significantly from children of responders, except that children of non‐responders were somewhat older (Appendix 1). Scars of 22 children with severe burns (79%) were assessed by observers; the other children were not willing to visit the outpatient clinic.

The median age of the included children was 2 years at burn (IQR = 1.0–3.0). Slightly more than half of the children (57%) were boys. Median %TBSA burned was 6% (IQR = 3–9), and median length of stay 5.0 days (IQR = 2.0–15.0). Most children (61%) did not undergo surgery. Scalds were most often (83%) the cause of burn and median time since burn was 5.5 years (IQR 5.4–5.8). The worst scar was often located on the arm (31%), trunk (23%) or leg (12%). The two subgroups (mild/intermediate vs. severe burns) differed significantly for most characteristics studied (Table 1). Of the twenty‐two children who visited the outpatient clinic, one child had both legs amputated, five children had persistent contractors, and one child had eyelid and mouth deformities.

**TABLE 1:** Characteristics of study sample

Variable	Total sample (*n* = 131)	Mild/intermediate burns (*n* = 103)	Severe burns (*n* = 28)	*p*‐value for difference
**Sex**: Male, *n*(%)	74 (56.5%)	56 (54.4%)	18 (64.3%)	0.348
**Age at survey**, median (IQR)	7.5 (6.5–8.8)	7.2 (6.5–7.8)	8.7 (7.6–10.1)	**0.001**
**Age at burn**, median (IQR)	2.0 (1.0–3.0)	2.0 (1.0–2.0)	2.0 (1.0–4.0)	0.412
**%TBSA burned**, median (IQR)	5.5 (2.9–9.0)	4.0 (2.0–6.0)	13.4 (11.3–17.6)	**<0.001**
**%TBSA full thickness**, median (IQR)	0.0 (0.0–0.5)	0.0 (0.0–0.0)	3.5 (0.0–8.0)	**<0.001**
**Length of hospital stay**, median (IQR)	5.0 (2.0–15.0)	3.0 (1.0–8.0)	21.5 (11.3–27.8)	**<0.001**
**Number of surgeries**, *n*(%)				**<0.001**
0	80 (61.1%)	73 (70.9%)	7 (25.0%)	
1	41 (31.3%)	29 (28.2%)	12 (42.9%)	
>1	10 (7.6%)	1 (1.0%)	9 (32.1%)	
**Reconstructive surgery**, *n*(%)	5 (3.8%)	0 (0.0%)	5 (17.9%)	**<0.001**
**Mechanical ventilation**, *n*(%)	1 (0.8%)	0 (0.0%)	1 (3.6%)	0.055
**Time since burn** (years), median (IQR)	5.5 (5.4–5.8)	5.5 (5.3–5.6)	6.5 (5.8–7.0)	**<0.001**
**Worst scar location**, *n*(%)				0.439
Head/face/neck	11 (8.4%)	6 (5.8%)	5 (17.9%)	
Trunk	30 (22.9%)	25 (24.3%)	5 (17.9%)	
Arm	41 (31.3%)	32 (31.1%)	9 (32.1%)	
Hand	9 (6.9%)	7 (6.8%)	2 (7.1%)	
Leg	16 (12.2%)	11 (10.7%)	5 (17.9%)	
Feet	9 (6.9%)	8 (7.8%)	1 (3.6%)	
Genitals	1 (0.8%)	1 (1.0%)	0 (0.0%)	
Buttocks	2 (1.5%)	2 (1.9%)	0 (0.0%)	
No scar	8 (6.1%)	8 (7.8%)	0 (0.0%)	
Missing	4 (3.1%)	3 (2.9%)	1 (3.6%)	
**Aetiology**, *n*(%)				0.577
Flame	13 (9.9%)	9 (8.7%)	4 (14.3%)	
Scald	109 (83.2%)	86 (83.5%)	23 (82.1%)	
Other	9 (6.9%)	8 (7.8%)	1 (3.6%)	

*Note*: Severe burns: >10% total body surface area (TBSA) burned if aged <10 years old at burn, >20% TBSA if aged ≥10 years old at burn, or more than 5% full thickness burns; *p*‐values in bold indicate statistically significant values.

### 3.2 Parent‐reported scar quality

**FIGURE 2:**
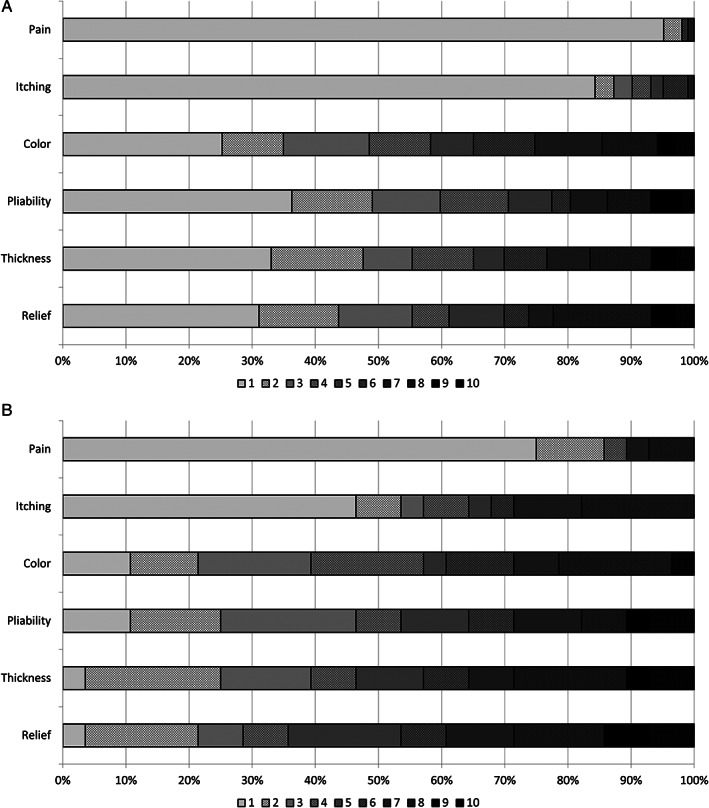
(A) Six POSAS characteristic scores in children with *mild/intermediate* burns 5–7 years after injury. With 1 corresponding to ‘no differences with normal skin’ and 10 to ‘very different to normal skin’. (B) Six POSAS characteristic scores in children with *severe* burns 5–7 years after injury. With 1 corresponding to ‘no differences with normal skin’ and 10 to ‘very different to normal skin’

The median parent‐reported POSAS score was 2.7 (IQR = 1.5–4.8) (Table 2). For seventeen children (13%) a score of 1.0 was reported, meaning that the parents did not notice any differences with normal skin. These were sixteen children with mild/intermediate burns and one with severe burns. Scars differed from normal skin for at least one characteristic in all other children. Median scores were highest for colour (4.0; IQR = 2.0–7.0) and relief (4.0; IQR = 1.0–7.0), and lowest for pain and itching (1.0 (IQR = 1.0–1.0). Scar quality of children with severe burns was rated worse than that of children with mild/intermediate burns. For children with severe burns, the median POSAS score was 4.2 (IQR = 2.2–5.9), which was 1.5 points higher than the score in the mild/intermediate group (*p* = 0.006). Except for colour, a significant higher score was reported in the severe group for all scar characteristics. In both subgroups, most differences compared to normal skin were reported for colour. Respectively, 75% and 89% of the scars of children with mild/intermediate and severe burns differed in colour compared to normal skin (Figure 2A,B). Children in this group who underwent surgery had a worse POSAS score compared to children who did not have surgery (median score 4.3 vs. 1.8; Appendix 2). For the majority of children with severe burns, differences on pliability (96%), thickness (96%), and relief (89%) were reported. Itching and pain were respectively reported for 54% and 25% of these children. Especially high scores were reported for pliability and thickness; a score of ≥8 was reported for 29% of the children (Figure 2B). Of the children with mild/intermediate burns, for 5% pain was reported, and for 16% itching. Pliability differed from normal skin in 64%, thickness in 67%, and relief in 69% of these children's worst scar (Figure 2A). Median POSAS scores ranged between 1.0 for pain and itching (IQR = 1.0–1.0) to 4.0 (IQR = 4.0–7.0) for colour. Children with severe burns who underwent surgery had a substantial poorer POSAS score (median score 4.8) compared to children who did not have surgery (median score 1.8; Appendix 2).

**TABLE 2:** Scar quality in children with mild/intermediate burns and severe burns 5–7 years postburn

POSAS items Patient Scale	Total sample (*n* = 128)		Mild and intermediate burns (*n* = 103)		Severe burns (*n* = 28)		*p*‐value for difference
	Median	25–75%	Median	25–75%	Median	25–75%	
**POSAS score**	2.7	1.5–4.8	2.7	1.4–4.5	4.2	2.2–5.9	**0.006**
Pain	1.0	1.0–1.0	1.0	1.0–1.0	1.0	1.0–1.8	**0.001**
Itching^a^	1.0	1.0‐1.0	1.0	1.0–1.0	2.0	1.0–7.0	**<0.001**
Colour	4.0	2.0–7.0	4.0	1.0–7.0	4.0	3.0–7.0	0.285
Pliability^a^	3.0	1.0‐6.0	3.0	1.0–5.0	4.0	2.3–7.0	**0.024**
Thickness	3.0	1.0–7.0	3.0	1.0–6.0	5.0	2.3–8.0	**0.012**
Relief	4.0	1.0–7.0	3.0	1.0–7.0	5.0	3.0–8.0	**0.009**
**Overall opinion**	4.0	2.0–6.0	3.0	1.0–6.0	5.0	2.3–7.0	0.052

*Note*: Severe burns: >10% total body surface area (TBSA) burned if aged <10 years old at burn, >20% TBSA if aged ≥10 years old at burn, or more than 5% full thickness burns; *p*‐values in bold indicate statistically significant values.

^a^ One missing value for a child in the mild/intermediate burn subgroup.

A median score of 4.0 (IQR 2.0–6.0) was reported for the parent's overall opinion of their child's worst scar (Table 2). The overall opinion did not significantly differ between the subgroups, though a trend was seen with higher scores reported in the severe group (5.0; IQR 2.0–7.0) compared to the mild/intermediate group (3.0; IQR 1.0–5.5) (*p* = 0.052). For 29 children (22%) the overall opinion item was scored as 1, meaning that the scar did not differ compared to normal skin according to the parents. Twenty‐six of these children had mild/intermediate burns. Children who underwent surgery had a worse overall opinion both in the subgroup of children with mild/intermediate burns (median score 5.5 vs. 2.0) and the subgroup of children with severe burns (median score 5.0 vs. 2.0) (Appendix 2).

### 3.3 Observer scores versus parent scores

The worst scar of the 22 children that visited the outpatient clinic was assessed by two observers (Appendix 3). Observer scores were 2.0 to 2.6 points lower than parent scores (*p* = 0.001–0.005), indicating that the observers evaluated the scars as being more comparable to normal skin than parents. The observers median scores ranged from 2.0 for colour, pliability, and thickness, to 3.0 for relief, whereas the median scores ranged from 4.0 for pliability to 5.5 for relief when assessed by the parents. The median overall opinion was also lower as scored by observers (median 3.0) than by parents (median 5.0) (*p* = 0.009).

### 3.4 Predictive factors of long‐term parent‐reported scar quality

Table 3 shows the univariate‐ and multivariate associated factors of reduced long‐term scar quality, for the POSAS score and the overall opinion score separately. Univariate predictive factors were identical for both outcomes, namely %TBSA burned, full thickness burns, length of hospital stay, number of surgeries, and functional site burns. Multivariate regression showed that number of surgeries was the only independent predictor for both outcomes. Children who had had surgery for their burn were thus more at risk for a worse scar quality.

**TABLE 3:** Prediction model for long‐term (5–7 post‐burn) POSAS score and overall opinion score

Variable	POSAS score						Overall opinion					
	Univariate regression			Multivariate regression			Univariate regression			Multivariate regression		
	Regression coefficient	SE	*p*‐value	Regression coefficient	SE	*p*‐value	Regression coefficient	SE	*p*‐value	Regression coefficient	SE	*p*‐value
Male gender	−0.005	0.349	0.989				0.065	0.458	0.887			
Age at injury	0.019	0.061	0.749				0.085	0.079	0.284			
%TBSA	0.067	0.024	**0.006**				0.079	0.032	**0.014**			
%TBSA full thickness	0.236	0.061	**<0.001**				0.270	0.079	**0.001**			
Length of hospital stay^a^	0.043	0.013	**0.001**				0.055	0.017	**0.001**			
Number of surgeries	0.675	0.128	**<0.001**	0.675	0.128	**<0.001**	0.865	0.169	**<0.001**	0.865	0.169	**<0.001**
Functional site burn vs. other	0.794	0.367	**0.033**				1.175	0.475	**0.015**			
Scalds vs. other	−0.636	0.459	0.169				−1.128	0.599	0.062			
Visible scar vs. other	0.161	0.629	0.799				0.161	0.819	0.845			
Time since burn	0.508	0.288	0.081				0.420	0.381	0.272			

*Note*: p‐values in bold indicate statistically significant values.

^a^ Length of hospital stay was highly correlated with %TBSA full thickness and %TBSA, and therefore not included in multivariate regression analysis.

## 4 DISCUSSION

This study evaluated the scar quality in children with burns 5–7 years after injury. The children's worst scar differed in 78% (overall opinion) to 87% (POSAS score) from normal skin. Colour differences were most often reported, whereas pain differences were least reported, possibly because neuropathic‐like sensations of children are hard to assess by parents.^8^ Except for colour, children with severe burns had a significantly higher score on all scar characteristics (representing poorer scar quality) compared to children with mild/intermediate burns. Parents of almost one out of three children (29%) in the severe burn group reported large differences compared to normal skin (POSAS ≥ 8) for pliability and/or thickness of their child's worst scar. Parent scores were on average 2.0 to 2.6 points higher compared to observer scores. Multivariate analysis showed that number of surgeries predicted both the POSAS score and the overall opinion score.

To the best of our knowledge, no other studies exist on long‐term scar quality in children with burns. A study by Goei et al. investigated scar quality on average 28 months after burns and included a subpopulation of children with a median TBSA of 5%.^7^ Outcomes slightly differed from our study. The median score on colour, pliability, thickness and relief was one point higher (representing a worse outcome) in our study. There were small differences in the study populations, and also time since injury and setting were different. Time since injury might have led to a changed coping behaviour, and the setting (hospital vs. home) to an unintended influence by the clinician and/or socially accepted answers. Shorter after burns, parents might view the scar in the light of the earlier phases during the evolution of the scar, whereas 5–7 years after injury they might be more aware that the final outcome is reached.

Pain and itching were scored very low; the median of both was one, which is equal to no pain/itching. Both pain and itching are common problems experienced by burn patients,^21^, ^22^, ^23^, ^24^, ^25^ which are also reported in the long‐term.^26^, ^27^, ^28^ However, an earlier study in children reported comparable low parent‐reported pain scores.^8^ This might be caused by the parent‐reported base of the study as neuropathic‐like sensations are hard to assess by parents.^8^ Similar results were found for anxiety; burned children reported substantially worse anxiety levels compared to their parent‐proxy outcomes.^29^ In our study, it is thus unsure whether pain (and itching) are indeed experienced by only few children, or that these symptoms were underestimated by their parents. Child self‐reported outcomes are important to fill this gap.

Parent‐reported outcomes differed on average at least two points from observer reported outcomes. This finding is in line with earlier studies that described that patient perspectives differ from observer perspectives.^4^, ^5^, ^8^ The large difference might be induced by the fact that the clinicians see the scar in the light of the trajectory over time and the improvement since burn injury, whereas the parent might look at the scar in the light of their child living the rest of his/her life with this scar, potentially incorporating more than pure scar quality in the evaluation of the scar. Parents might therefore find the scar quality substantially worse compared to the clinicians. This highlights the importance of patient‐reported outcomes and the importance of a valid instrument to assess paediatric scar quality by children themselves. Furthermore, an earlier study showed that when patients rate the severity of their scar, treatment is more likely to fit the patients' needs.^6^ It is thus very important to incorporate patient evaluation in paediatric scar treatment, if possible, not only by the parent but also by the child him/herself, as their opinions may differ too.

Multivariate analysis showed that number of surgeries was the only independent predictive factor for both POSAS and overall opinion score. This is in line with earlier studies that also found more surgeries to be a predictor of worse short‐term scar quality.^9^, ^12^ Children who had had surgery for their burn are thus more at risk of worse scar quality. Not surgery in itself, but as a proxy of burn severity, is a predictor for worse scar quality. Deep burns likely have a worse outcome if treated without surgery, so our results are not a reason to avoid surgery. Earlier studies have clearly shown that avoiding surgery in deep burns result in worse scarring.^30^, ^31^ Another proxy measure of burn severity; a greater burn size (%TBSA burned), also predicted a reduced short‐term scar quality in previous studies.^4^, ^8^, ^9^ In univariate analyses, %TBSA and other indicators of burn severity (full thickness burns and length of hospital stay) were found to be associated, but not in multivariate analyses. Results on whether full thickness burns is a predictor are inconsistent, an earlier study found that partial thickness burns were associated with a better scar quality,^8^ whereas another study did not find this result.^12^ Our study also showed that scarring on a functional site predicted scar quality in univariate analyses. As far as we know, this factor has not yet been studied as a predictor before. Nevertheless, in clinical practice children with scars on a functional site seem to experience more contractures and a poorer scar quality, possibly due to a scar on a functional site being more often stretched. The findings of our study that children's age, sex and aetiology of the injury were not associated with scar quality are in line with earlier studies.^8^, ^9^, ^12^

In the vast majority of children with mild/intermediate burns and severe burns, their worst scar differed from normal skin 5–7 years post‐burn. Informing children and parents about the final outcome is very important. An earlier study showed that many children expect that their scar will look as normal skin after scar maturation.^2^ Also, a recent review reported that children and parental concern, and appearance are problems that are prevalent in the long‐term.^3^ It is thus important to counsel both children and their parents on the expectations of the final outcome of their (children's) scar(s). This is important for those with severe burns and who needed surgery as they were at a higher risk of a poorer long‐term scar quality, but also other children and their parents should be counselled on realistic expectations.

This study contains strengths and limitations. Strengths include the multicentre approach, the relatively large sample size, the small amount of missing data, and the small differences between responders and non‐responders. Another strength is the use of the POSAS instrument; it is validated, includes all most relevant scar characteristics, and is the most frequently used scale.^32^, ^33^, ^34^, ^35^ A limitation was our inability to study other potential predictors, like skin type^12^ and time to wound healing.^4^, ^9^, ^10^, ^11^, ^13^, ^14^, ^36^ Also, in children with multiple scars, only the worst scar was evaluated, which might have led to a slight overestimation of how scars differed from normal skin. Another limitation is the relatively small sample size to develop robust prediction models. For good prediction modelling large numbers are needed.^37^, ^38^ However, it is hard to collect large sample sizes in burns and our sample was relatively large for burns.^39^ If available, combining existing datasets might overcome this problem.^40^ Other limitations include the lack of information regarding scar treatment, which may have influenced scar outcome, and the fact that parent‐proxy outcomes were used instead of children's own evaluation. Outcomes, in particular pain and itching, might be scored differently if children evaluated them themselves. However, to the best of our knowledge, no paediatric scar quality assessment instrument was available at time of study.

## 5 CONCLUSION

Five to seven years after burns, the worst scar differed in most children from normal skin, with most and largest differences reported for colour. Children with severe burns had significantly higher scores on all scar characteristics, except for colour compared with children with mild/intermediate burns. Children who needed surgery for their burn(s) were at a higher risk of having a poorer long‐term scar quality. These insights are useful in the counselling of children and their parents on the expectations of the final outcome of their (children's) scar(s). And, these insights help in further targeting scar prevention strategies for the individual child.

## CONFLICT OF INTEREST

No conflicts of interest to disclose.

## Supporting information


**Appendix S1**: Supporting information
