# Supplementary material for: Scar quality in children with burns 5–7 years after injury: A cross‐sectional multicentre study
Source: Wound Repair Regen. 2021 Jun 16;29(6):951–60. doi: 10.1111/wrr.12953 (PMC8596883; doi:10.1111/wrr.12953)
Supplement: Supplementary file 1 — Appendix S1: Supporting information [file WRR-29-951-s001.pdf]

Supplementary materials

Appendix 1. Characteristics of responders versus non-responders

**Variable**	**Responders (n=131)**	**Non-responders (n=130)**	**p-difference between subgroups**
**Sex: Male**, n(%)	74 (56.5%)	75 (57.7%)	0.844
**Age at burn**, median (IQR)	2.0 (1.0-3.0)	2.0 (1.0-4.0)	**0.038**
**%TBSA burned**, median (IQR)	5.5 (2.9-9.0)	4.0 (2.0-12.3)	0.105
**%TBSA full thickness**, median (IQR)	0.0 (0.0-0.5)	0.0 (0.0-0.3)	0.571
**Length of hospital stay**, median (IQR)	5.0 (2.0-15.0)	4.0 (2.0-12.3)	0.381
**Number of surgeries**, n(%)			0.191
0	80 (61.1%)	89 (68.5%)	
1	41 (31.3%)	28 (21.5%)	
>1	10 (7.6%)	13 (10.0%)	
**Reconstructive surgery**, n(%)	5 (3.8%)	7 (5.4%)	0.568
**Etiology**, n(%)			0.687
Flame	13 (9.9%)	17 (13.1%)	
Scald	109 (83.2%)	103 (79.2%)	
Other	9 (6.9%)	10 (7.7%)	

Note: p-values in bold indicate statistically significant values.

Appendix 2. Parent-reported scar quality according to surgery and severity of burns

	Severity of burns			
	Mild/intermediate burns		Severe burns	
Surgery	POSAS score, median (25-75%)	Overall opinion, median (25-75%)	POSAS score, median (25-75%)	Overall opinion, median (25-75%)
No surgery	1.8 (1.2-3.4)	2.0 (1.0-5.0)	1.8 (1.5-2.2)	2.0 (1.0-3.0)
Surgery	4.3 (2.6-5.7)	5.5 (5.0-7.0)	4.8 (3.5-6.5)	5.0 (5.0-8.0)

Note. The number of children per subgroup range between n=7 (severe burns and no surgery) and n=73 (mild/intermediate burns and no surgery).

Appendix 3. Parent-reported and observer-reported scar quality in children with severe burns 5-7 years postburn (n=22)

POSAS items	Patient scale		Observer scale		Patient score – observer score	p-value
	Median	25-75%	Median	25-75%	Difference	Difference
**POSAS score**	4.9	2.7-7.4	2.3	1.7-3.2	2.6	0.001
Color	4.0	2.8-7.3	2.0	1.5-3.0	2.0	0.002
Pliability	4.0	2.3-7.3	2.0	2.0-3.3	2.0	0.005
Thickness	4.5	2.0-8.0	2.0	1.8-3.0	2.5	0.005
Relief	5.5	2.3-8.0	3.0	2.0-4.0	2.5	0.003
**Overall opinion**	5.0	2.0-8.0	3.0	2.0-4.0	2.0	0.009
